# Evolving understanding of the type VII secretion system in Gram-positive bacteria

**DOI:** 10.1371/journal.ppat.1010680

**Published:** 2022-07-28

**Authors:** Brady L. Spencer, Kelly S. Doran

**Affiliations:** Department of Immunology and Microbiology, University of Colorado School of Medicine, Aurora, Colorado, United States of America; University of Geneva Faculty of Medicine: Universite de Geneve Faculte de Medecine, SWITZERLAND

## Introduction to Gram-positive T7SSb

**Fig 1:**
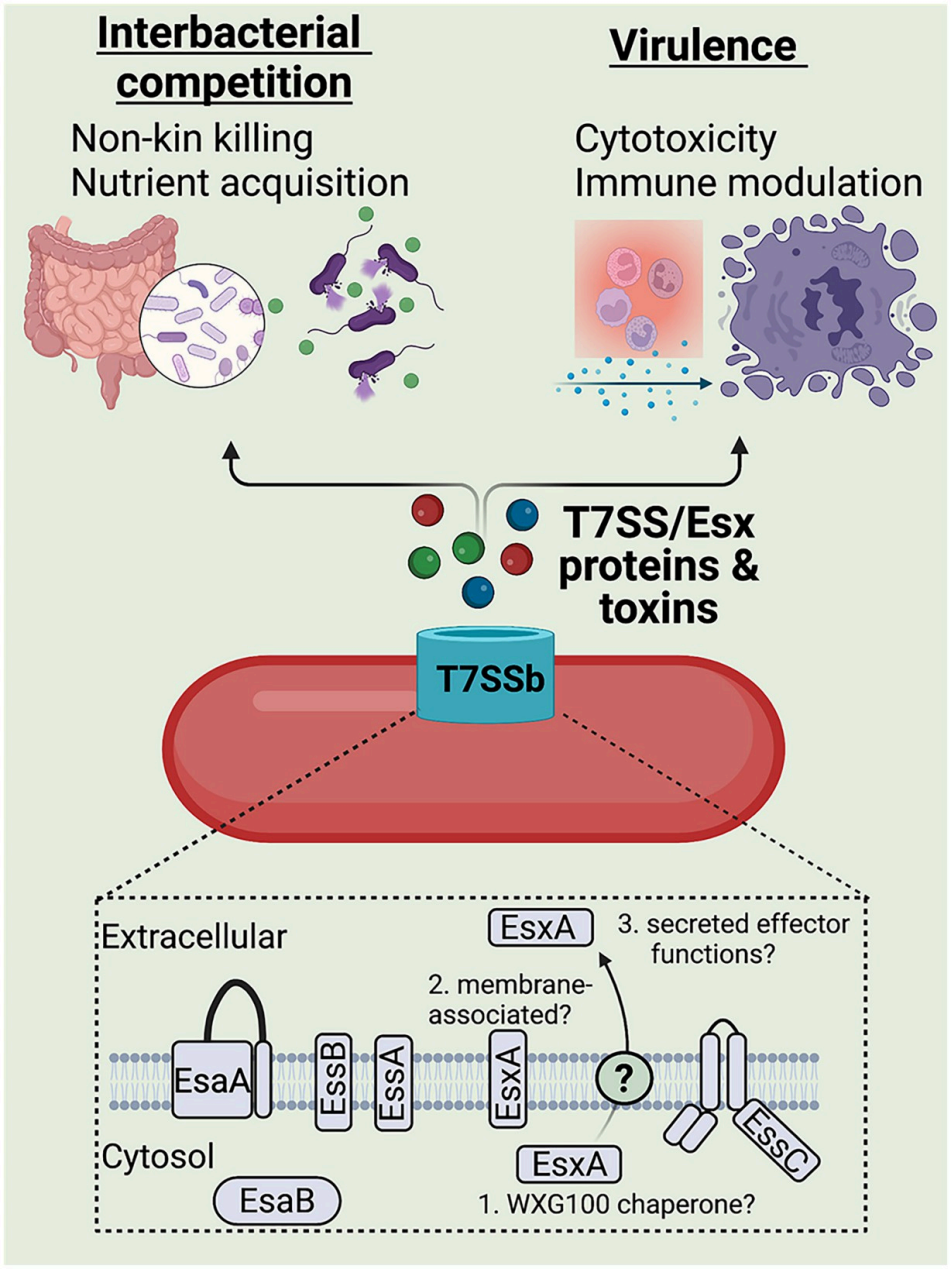
The T7SSb of Gram-positive bacteria encode primarily membrane-bound machinery including an EssC ATPase, which facilitates the export of small alpha-helical proteins and toxins. Structural depictions of T7SS machinery proteins as described in [2]. The putative functions of EsxA in T7SSb require further investigation but EsxA may putatively (1) chaperone other T7SS substrates; (2) associate with membrane T7SSb core components; and (3) exhibit cytotoxic and pore-forming activity as secreted effector. While T7SSb substrates are still being elucidated and characterized, the T7SSb as a whole has been shown in many species to play roles in interbacterial competition and virulence.

Secretion systems are commonly used by bacteria to export proteins to their extracellular environment and are integral in bacterial niche adaptation. While most secretion systems are encoded by Gram-negative organisms, the type VII secretion system was discovered in Actinobacteria and then in Firmicutes (designated T7SSa and T7SSb, respectively) and has been implicated in development, interbacterial competition, nutrient acquisition, and virulence (Fig 1) [1]. Broadly, the T7SS is characterized by ATPase-driven export of approximately 100-residue alpha-helical proteins that lack traditional signal sequences but contain a central WXG motif, or “WXG100” proteins, with EsxA being the first identified T7SS substrate [1]. The T7SSb core machinery required for substrate secretion has been best characterized in *Staphylococcus aureus* and includes cytoplasmic EsaB as well as membrane-bound EsaA, EssA, EssB, and EssC (the ATPase that powers substrate translocation and confers substrate specificity [2]; Fig 1). Interestingly, WXG100 protein EsxA has been hypothesized to perform dual roles as both a secreted effector and as a core T7SSb component, as it may associate with T7SSb membrane proteins [2] and/or may chaperone other T7SSb substrates (as proposed for WXG100-like proteins in [3]). However, further experimental investigation is needed to confirm these putative EsxA functions (Fig 1). Translocation of *S*. *aureus* T7SS substrates also requires peptidoglycan hydrolase EssH, which may facilitate apparatus assembly [4]. However, while a T7SSb membrane complex has been observed [2], the biochemical functions of these proteins (aside from EssC) are still being elucidated. The structure of these components has been nicely reviewed elsewhere [2,5]. Homologs of these T7SSb core components have now been described in numerous other Gram-positive organisms, including *Bacillus*, *Listeria*, *Enterococcus*, and *Streptococcus* [1,2,5]. Here, we review the known and recently discovered functions of the T7SSb in host and bacterial interactions as well as discuss hurdles in the study of T7SSb biology.

## Type VII secreted factors: What has been shown to date?

The T7SSb encodes extensive inter- and intraspecies heterogeneity in its putative secreted substrates [2,6]. While most T7SS substrates are encoded within the T7SS locus, other orphaned effectors exist including LXG toxins and WXG100 proteins and some T7SS substrates may not yet be identified. This diversity of secreted toxins/effectors may result in advantages for certain strains during host colonization and/or pathogenesis. Because these substrate repertoires vary extensively, T7SS variants have been designated within species based on differences in their EssC C-terminus and associated downstream effectors. For example, bioinformatic analyses of T7SSb in Firmicutes have designated four T7SS variants in Group B *Streptococcus* (GBS) and *S*. *aureus* [2,7] and seven variants in *Listeria monocytogenes* [6]. While EsxA secretion has been demonstrated in numerous Firmicutes, additional T7SSb-secreted effectors have been confirmed in *S*. *aureus* and *Streptococcus intermedius*, including staphylococcal WXG100 protein EsxB, small alpha-helical proteins EsxC and EsxD, LXG toxins TspA and EsxX, and nuclease toxin EsaD, as well as *S*. *intermedius* LXG toxins TelA, TelB, and TelC [2,5]. While these effectors share minimal sequence homology across T7SSb subtypes and species, WXG100 proteins, WXG100-like SACOL2603 proteins, other small alpha-helical proteins, and LXG toxins are commonly encoded downstream of EssC in T7SSb loci and may share similar functions across systems [2]. Thus, more work is needed to confirm secretion of these putative effectors in other Gram-positive organisms to understand their unique contributions to bacterial fitness and host–pathogen interactions.

## Functions of the T7SSb: Interbacterial competition and/or accidental virulence?

The T7SSb has been shown to mediate interbacterial competition in Firmicutes and relies on biochemically diverse toxins encoding an “LXG” motif [2,5,8]. Protection from killing is conferred via expression of cognate immunity factors. Eloquent studies have been performed in *S*. *aureus* implicating nuclease EsaD in interbacterial competition and downstream immunity factor EsaG in protecting from this killing [2]. Similarly, in *Enterococcus faecalis*, the T7SSb promoted killing of *Enterococcus faecium*, *S*. *aureus*, and *L*. *monocytogenes* upon phage induction of T7SS (likely via OG1RF_11121, an LXG toxin with unknown function), but did not promote killing of any streptococcal strain nor any Gram-negative organism tested [9]. Additionally, *Bacillus subtilis* encodes five toxic LXG proteins (including ribonuclease YxiD), which may promote the observed T7SSb-dependent *B. subtilis* killing of *Lactococcus lactis* [8]. Finally, in *S*. *intermedius*, LXG toxin TelC (a lipid II phosphatase) mediated killing against *S*. *intermedius* strains, *Streptococcus pyogenes*, and *E*. *faecalis*, but not against the Gram-negative organisms tested [3]. In the above examples, LXG toxin-mediated killing could be inhibited by an adjacently encoded immunity factor. Most recently, *Streptococcus suis* has been shown to encode three putative LXG proteins of unknown function, which may mediate observed T7SSb-dependent intraspecies killing [10]; however, direct LXG killing remains to be shown. In addition to bacterial killing, nutrient acquisition is also integral for interbacterial competition. Recently, the T7SSb of rhizobacterium *Bacillus velezensis* was shown to promote iron acquisition and root colonization [11], and the *S*. *aureus* T7SSb is regulated by iron availability [2]; thus, the role for T7SSb in nutrient acquisition may be underappreciated. Because of these functions and because the T7SSb also promotes functions such as development of *Streptomyces scabies* [5], it is hypothesized that the T7SSb may have evolved for housekeeping roles and/or bacterial competition and niche establishment [2].

T7SSb also clearly promotes pathogenesis in many bacterial species [1], even if this virulence contribution may be evolutionarily accidental. In *S*. *aureus*, the T7SSb and secreted effectors (EsxA, EsxB, and EsxC, as well as toxins EssD/EsaD, TspA, and EsxX) have been implicated in virulence in models of kidney abscess, pneumonia, and systemic infection in mice and zebrafish [2]. T7SSb in other species also promotes host virulence, such as during GBS meningitis, *Bacillus anthracis* and *S*. *suis* systemic infection, as well as *Streptococcus gallolyticus* promotion of gut colonization and colon tumor development [5,7,12,13]. Staphylococcal and enterococcal T7SSb further promote host persistence in the nasopharynx and the vaginal tract, respectively [2,14]. In contrast, T7SSb in *Listeria* strain EGDe reduced virulence potential [15], possibly due to that strain’s specific T7SSb effector repertoire. While more work is needed to elucidate species-specific T7SSb mechanisms of virulence, T7SSb is known in some instances to modulate host inflammatory and cell death pathways. During experimental GBS meningitis, the T7SSb promoted increased levels of neutrophil chemoattractant KC and promoted cell death in brain endothelium, and this was dependent on WXG100 protein EsxA [7]. Interestingly, in *S*. *aureus*, T7SSb promotes resistance to host fatty acids [16], as well as modulates cytokine responses and dampens macrophage recruitment during murine blood infection [17], but not in zebrafish [18]. Thus, T7SSb-driven immune responses or evasion may depend on the specific effectors secreted by that strain and/or differential regulation of T7SSb loci.

## Hurdles to study of the T7SSb: Which substrates are secreted and when?

In addition to T7SSb effector diversity, regulation of this locus in various niches likely impacts T7SS-dependent phenotypes. Staphylococcal T7SS regulation has been extensively studied and is quite complex involving two-component systems (TCS), single component regulators, and sRNA regulation (see excellent comprehensive review [2]). In other Firmicutes, TCS and single component regulators modulate T7SSb expression. For example, in *B*. *subtilis*, T7SS was regulated by the DegS-DegU TCS [1]; in *E*. *faecalis*, phage infection induction of T7SSb required serine/threonine kinase IreK and a GntR-family regulator [9]; and in GBS, deletion of *cas9* up-regulated T7SSb genes [19]. Generally across species, the T7SSb appears to be induced *in vivo*, in host-mimicking conditions (pulmonary surfactant, serum, fatty acids [2]), and/or during cell stress (phage infection and cell membrane-targeting antibiotic daptomycin in *E*. *faecalis* [9]). Using RNA-seq data generated from a study examining a spectrum of stress conditions across numerous bacterial species [20], we have observed that GBS *esxA* is induced upon oxygen and nutrient deprivation and in the presence of serum. Further analysis of this dataset is warranted to assess T7SS induction in other Firmicutes, which may help identify *in vitro* inducing conditions for future T7SS studies.

Despite these observations, T7SSb-inducing conditions can be extremely species and even strain specific. Consequently, only a handful of T7SSb effectors have been confirmed to date and most of these were identified in *S*. *aureus* via targeting of predicted effectors, likely based on T7SS locus proximity and presence of T7SS-associated motifs. Recent studies have employed a broader approach to identify a wider scope of T7SSb effectors using mass spectrometric/proteomic analysis [3,18]. Interestingly, putative T7SS effectors encoded elsewhere in the genome were identified, including orphaned LXG toxins TspA (*S*. *aureus*) and TelC (*S*. *intermedius*). This approach increases the scope of potential T7SSb effector discovery; yet, identification of additional effectors may still be limited by low expression/repression of T7SSb *in vitro*. Thus, employing a combination of host-like inducing conditions along with a broader mass spectrometry/proteomics approach may allow for *in vitro* T7SS expression, activity, and subsequent elucidation of the full repertoire of T7SSb effectors in a given strain or species.

## Conclusions

While there is still much to learn, the evidence thus far clearly indicates a role for the Gram-positive T7SSb system in both interbacterial competition and virulence. However, it is evident that extensive heterogeneity exists in the T7SSb among genera, species, and strains. We propose that these T7SSb-mediated phenotypes may depend on T7SSb subtype-specific regulation and secreted effectors, and that this T7SSb-associated diversity may promote selection of certain strains/species in specific environments. We further hypothesize that T7SSb allows Gram-positive bacteria, particularly opportunistic pathogens, to readily adapt to their environment and promote their survival, whether during colonization or disease pathogenesis. Continual improvement in the identification of T7SSb-inducing conditions and effectors will help determine the full contribution of T7SSb to the survival strategies of Gram-positive bacteria.
